# Nearside-Farside Analysis of the Angular Scattering for the State-to-State H + HD → H_2_ + D Reaction: Nonzero Helicities

**DOI:** 10.1021/acs.jpca.1c06195

**Published:** 2021-09-22

**Authors:** Chengkui Xiahou, J. N. L. Connor

**Affiliations:** † School of Pharmacy, Qilu Medical University, Zibo Economic Zone, Zibo City 255300, Shandong, People’s Republic of China; ‡ Department of Chemistry, The University of Manchester, Manchester M13 9PL, United Kingdom

## Abstract

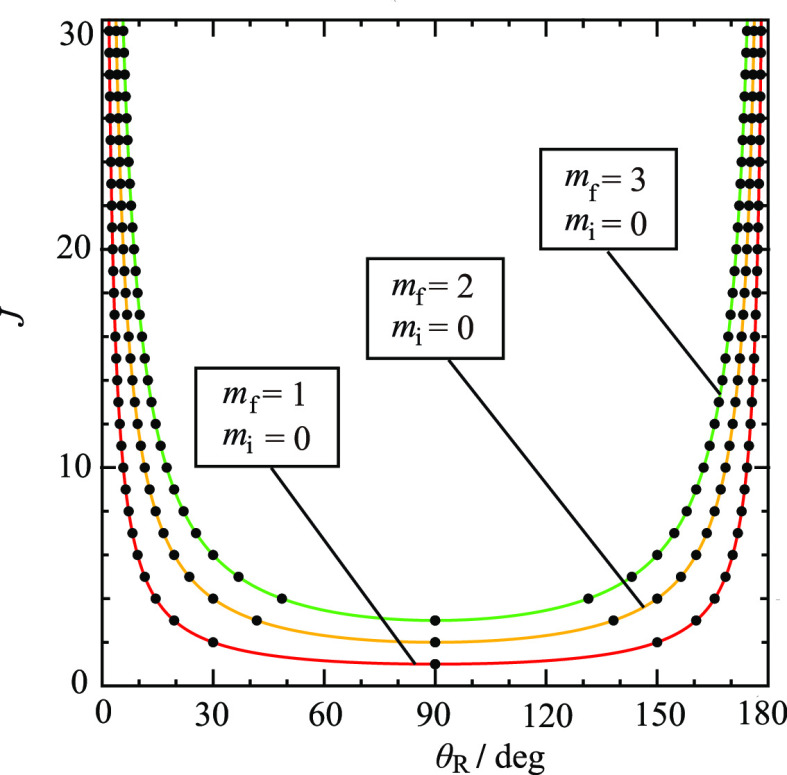

We theoretically analyze the differential cross sections (DCSs) for the state-to-state reaction, H + HD(*v*_i_ = 0, *j*_i_ = 0, *m*_i_ = 0) → H_2_(*v*_f_ = 0, *j*_f_ = 1,2,3, *m*_f_ = 1,..,*j*_f_) + D, over the whole range of scattering angles, where *v*, *j*, and *m* are the vibrational, rotational, and helicity quantum numbers for the initial and final states. The analysis extends and complements previous calculations for the same state-to-state reaction, which had *j*_f_ = 0,1,2,3 and *m*_f_ = 0, as reported by XiahouC.; ConnorJ. N. L.Phys. Chem. Chem. Phys.2021, 23, 13349–133693409693410.1039/d1cp00942g. Motivation comes from the state-of-the-art experiments and simulations of Yuan et al.Nature Chem.2018, 10, 653–65829686377 who have measured, for the first time, fast oscillations in the small-angle region of the degeneracy-averaged DCSs for *j*_f_ = 1 and 3 as well as slow oscillations in the large-angle region. We start with the partial wave series (PWS) for the scattering amplitude expanded in a basis set of reduced rotation matrix elements. Then our main theoretical tools are two variants of Nearside-Farside (NF) theory applied to six transitions: (1) We apply unrestricted, restricted, and restrictedΔ NF decompositions to the PWS including resummations. The restricted and restrictedΔ NF DCSs correctly go to zero in the forward and backward directions when *m*_f_ > 0, unlike the unrestricted NF DCSs, which incorrectly go to infinity. We also exploit the Local Angular Momentum theory to provide additional insights into the reaction dynamics. Properties of reduced rotation matrix elements of the second kind play an important role in the NF analysis, together with their caustics. (2) We apply an approximate N theory at intermediate and large angles, namely, the Semiclassical Optical Model of Herschbach. We show there are two different reaction mechanisms. The fast oscillations at small angles (sometimes called Fraunhofer diffraction/oscillations) are an NF interference effect. In contrast, the slow oscillations at intermediate and large angles are an N effect, which arise from a direct scattering, and are a “distorted mirror image” mechanism. We also compare these results with the experimental data.

## 1 Introduction

The H + H_2_ → H_2_ + H reaction and its isotopic variants are important benchmarks in the theory of chemical reaction dynamics. In particular, measurements and calculations of state-to-state differential cross sections (DCSs) can provide detailed information on the dynamics and mechanism of this class of reactions.

Recently, an important experimental advance has been reported by Yuan et al.^1^ for the H + HD → H_2_ + D reaction. They have measured, for the first time, *fast oscillations* in the small-angle region of the degeneracy-averaged DCSs (abbreviated as daDCSs). They reported daDCSs for the following two transitions.
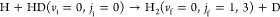
R1In reaction (R1), *v*_i_, *j*_i_ and *v*_f_, *j*_f_ are the initial and final vibrational and rotational quantum numbers of the diatomic molecules, respectively. The experiment of Yuan et al.^1^ used a high-resolution molecular-beam apparatus, crossed at 150°, with a velocity map imaging product detection at a translational energy of 1.35 eV. These daDCS measurements are the current state-of-the-art. Related experimental work can be found in refs (2−5).

The purpose of the present paper is to analyze and quantitatively understand the daDCSs of Yuan et al.^1^ To do this, we start with the *helicity* (or *body-fixed*) representation for the scattering amplitude. We then have to consider the following 16 state-to-state DCSs
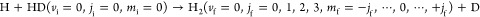
R2where *m*_i_ and *m*_f_ are the helicity quantum numbers for the initial and final states, respectively.

In our earlier paper^6^ (denoted XC1), which is a companion to this one, we studied theoretically the DCSs for the four state-to-state transitions in reaction (R2) with *m*_f_ = 0, namely
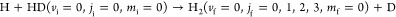
R3In particular, we analyzed the dynamics of the angular scattering for reaction (R3) in order to understand the physical content of the structure in the four helicity-resolved DCSs.^6^ We discovered: glory scattering at small angles, broad or “hidden” nearside rainbows, Nearside-Farside (NF) interference effects (sometimes called Fraunhofer diffraction/oscillations), a “CoroGlo” test to distinguish corona and forward glory scattering, and a “distorted mirror image” mechanism present at intermediate and large angles.^6^

In this paper, we focus on the DCSs for state-to-state transitions with *nonzero helicities*. This reduces the number of DCSs in reaction (R2) to 12. A further reduction is possible because the DCSs for *m*_f_ = −1, −2, −3 are equal to those for *m*_f_ = +1, +2, +3, respectively. This leaves the following six DCSs to be analyzed.
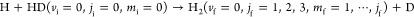
R4We will often write 000 → 011, 000 → 021, 000 → 031, 000 → 022, 000 → 032, and 000 → 033 for the six transitions or, more simply, 011, 021, 031, 022, 032, and 033.

There is a fundamental difference between DCSs with *m*_f_ = 0 and those with *m*_f_ > 0 for reactions of the type (R3) and (R4). All DCSs with *m*_f_ > 0 are identically equal to zero in the forward (θ_R_ = 0°) and backward (θ_R_ = 180°) directions in the center-of-mass reference frame, which is a consequence of the conservation of angular momentum. Furthermore, the partial wave series (PWS) for the scattering amplitude for *m*_f_ = 0 uses a basis set of *Legendre polynomials*, whereas for *m*_f_ > 0 the basis set consists of *reduced rotation matrix elements* (also called *Wigner* or *little d functions*), which simplify to associated Legendre functions when *m*_i_ = 0. This means the theoretical analysis is more complicated and difficult for the *m*_f_ > 0 case compared to *m*_f_ = 0.

Now there has been one previous NF analysis of DCSs for chemical reactions with *m*_f_ > 0, which was made more than 20 years ago.^7^ In this work, Dobbyn et al.^7^ made the following important observation (on page 1117):

“...although the PWS becomes more complicated for more general types of collisions, this has little impact on the physical insight provided by a NF analysis”.

Thus, in this paper, (two variants of) NF theory will be used to provide physical insight into the reaction dynamics. Note that the NF theory was used extensively in XC1.^6^ In particular, an NF analysis has the advantage that the semiclassical (asymptotic) picture is still evident, even though semiclassical techniques, such as the stationary phase or saddle point methods, are not applied. Note that Yuan et al.^1^ have conjectured on the role an NF analysis plays in explaining oscillatory structures in their DCSs. The two NF theories we use are

(1) For a PWS with a basis set of Wigner functions, we use three NF decompositions: *unrestricted* (^unres^NF),^8^*restricted* (^res^NF),^9,10^ and *restrictedΔ* (^resΔ^NF).^7^ The ^unres^NF decomposition is a straightforward generalization^8^ of the NF decomposition for a Legendre PWS.^8,11^ The ^unres^NF DCSs incorrectly diverge as θ_R_ → 0°, 180°. In contrast, the ^res^NF and ^resΔ^NF DCSs correctly go to zero as θ_R_ → 0°, 180°.^7,9,10^
  The properties of the *caustics* of Wigner functions as well as those for *reduced rotation matrix elements of the second kind* play an important role in the definitions of ^res^NF and ^resΔ^NF.^7,9,10^ We also perform a *resummation* for a PWS of Wigner functions,^12^ since it is well-known that a resummation can improve the physical effectiveness of an NF decomposition.^13−19^ In fact, the present paper is the first time that resummation theory has been combined with the ^res^NF and ^resΔ^NF decompositions.
  The above remarks apply, in particular, to NF analyses of the full DCSs for the six transitions. We also report the results (including resummations) of the ^unres^NF decomposition for the *Local Angular Momentum* (LAM), since this provides important additional insights into the reaction dynamics.^13−16^
(2) A simple approximate N model, the *Semiclassical Optical Model* (SOM), which was originally introduced by Herschbach.^20,21^ It is particularly useful for understanding structures in a DCS at intermediate and large angles for direct reactions.^6,7^

This paper is organized as follows: Section 2 summarizes the partial wave theory and explains our conventions and definitions for the DCSs and LAMs. This section also includes a discussion of the caustic properties that we need and summarizes the ^unres^NF, ^res^NF, and ^resΔ^NF decompositions. Section 3 outlines the resummation for a PWS of Wigner functions. The properties of the input scattering matrices for the six transitions are presented in Section 4; we use the same accurate scattering matrix elements employed by Yuan et al.^1^ in a simulation of their experiments. In Section 5, we discuss in detail the behavior of the ^unres^NF, ^res^NF, and ^resΔ^NF DCSs at small and large angles, as this has not been done before. Our results for the full and NF DCSs and LAMs, including resummations, are presented and discussed in Sections 6 and 7, respectively. The SOM DCSs at intermediate and large angles are presented and discussed in Section 8. We report daDCSs in Section 9, where we make comparisons with the experimental data. Our conclusions are in Section 10. Most of our results are presented graphically.

Appendix A proves that the state-to-state *m*_i_ = 0 DCSs for *m*_f_ = −1, −2, −3 and *m*_f_ = +1, +2, +3 are equal, respectively. In applications of the NF theory, it is essential to use unambiguous and consistent definitions for the special functions (of the first and second kinds) employed in the various NF decompositions. In Appendix B, we gather together the precise mathematical definitions that we use, since there is often more than one definition in the literature.

We also emphasize the following: This paper complements and extends XC1,^6^ where additional discussions and references can be found. These two papers illustrate the potency of the NF theory for divers applications.

## 2 Partial Wave Theory

### 2.1 Partial Wave Series

We start with the helicity (or body-fixed) PWS representation of the scattering amplitude for reaction (R4) at a fixed translational (or total) energy^22,23^
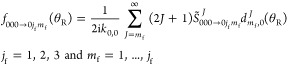
1where *k*_0,0_ is the translational wavenumber for relative motion in the initial channel, *J* is the total angular momentum quantum number, *S̃*_000→0*j*_f_*m*_f__^*J*^ is a modified scattering matrix element, and *d*_*m*_f_,0_^*J*^(θ_R_) is a reduced rotation matrix element (also called a Wigner function or “*little d*” function) as defined by Edmonds.^24^ The reactive scattering angle θ_R_ is the angle between the incoming H atom and the outgoing H_2_ molecule in the center-of-mass reference frame. Thus, θ_R_ = 0° and θ_R_ = 180° define the forward and backward directions, respectively. In practice, the upper limit of *J* = ∞ in the PWS is replaced by a finite value, *J* = *J*_max_. This assumes that all partial waves with *J* > *J*_max_ can be neglected. In our applications, there are ∼40 numerically significant coherent partial waves, which makes the direct physical interpretation of the PWS very difficult or impossible. In addition, a constant phase has been omitted from eq 1.

The corresponding state-to-state DCS is given by
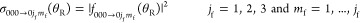
2The PWS representation (1) is also valid for *m*_f_ = 0, as further analyzed in detail for the H + HD reaction in XC1;^6^ however, DCSs with *m*_f_ = 0 will only be needed in Section 9, when we discuss daDCSs. In passing, we note that the PWS (1) remains valid for *m*_f_ < 0, provided the starting value of the summation is replaced by *J* = |*m*_f_|. This is only needed in Appendix A. In the remainder of this paper, we will often drop the channel labels from *f*, *k*, *S̃*, σ, etc. to keep the notation simple. We will also write *S̃*_*J*_ ≡ *S̃*^*J*^.

To provide additional insight into the reaction dynamics, we also perform a *Local Angular Momentum* analysis.^13−16^ The LAM analysis provides information on the total angular momentum variable that contributes to the scattering at an angle θ_R_ under semiclassical conditions. It is defined by
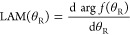
3Note that the arg in eq 3 is not necessarily the principal value in order that the derivative be well-defined.

Next we describe the *unrestricted NF decomposition* for the full PWS (1), which is the simplest NF decomposition, and we point out a limitation when *m*_f_ > 0.

### 2.2 Unrestricted Nearside-Farside Decomposition (^unres^NF)

We *exactly* decompose *f*(θ_R_) by writing it as the sum of two subamplitudes N and F, namely^8^
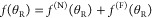
4This is accomplished by *exactly* decomposing the *d*_*m*_f_,0_^*J*^(θ_R_) in eq 1 into *traveling angular functions of degree J and order m*_f_
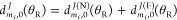
5where, for θ_R_ ≠ 0,π
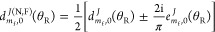
6In eq 6, the *e*_*m*_f_,0_^*J*^(θ_R_) are *reduced rotation matrix elements of the second kind* (also known as “*little e*” functions) and defined in Appendix B. We see in eq 6 that the *d*_*m*_f_,0_^*J*(N,F)^(θ_R_) are linear combinations of reduced rotation matrix elements of the first and second kinds or, equivalently, from Appendix B, a linear combination of Jacobi functions of the first and second kinds.

Using eqs 4–(6), the N and F subamplitudes are given by (θ_R_ ≠ 0,π)
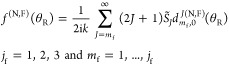
7which is called the *unrestricted NF decomposition*. The adjective “unrestricted” is added because eq 7 can be used for all θ_R_ ∈ (0,π) with no restriction on the sum over *J*. We also sometimes write ^unres^NF. The corresponding N and F DCSs are given by
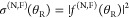
8With the help of eqs 2 and (8), we obtain
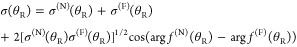
9Equation 9 is the *Fundamental Identity for Full and NF DCSs* and is exact.^25^

Similarly, we can define (unrestricted) N and F LAMs
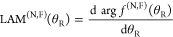
10There is an exact *Fundamental Identity for Full and NF LAMs* analogous to eq 9, although more complicated in form.^25^ As before, the args in eq 10 are not necessarily principal values in order that the derivatives be well-defined.

**Figure 1:**
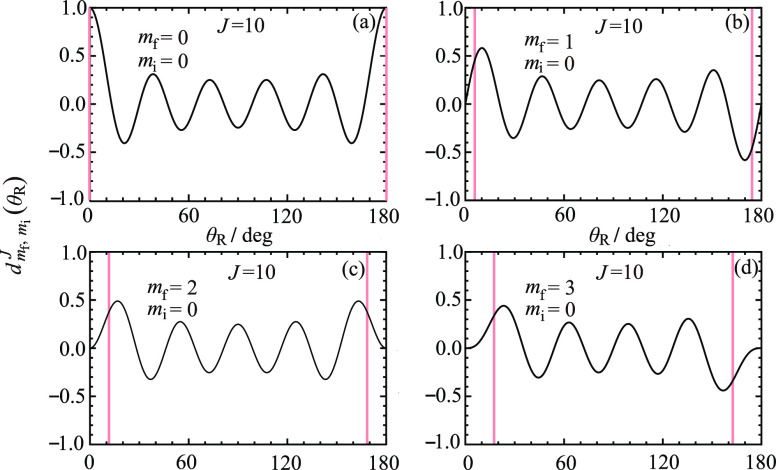
Plots of *d*_*m*_f_, *m*_i__^*J*^ (θ_R_) vs θ_R_/deg for *J* = 10, *m*_i_ = 0 (a) *m*_f_ = 0, (b) *m*_f_ = 1, (c) *m*_f_ = 2, (d) *m*_f_ = 3. The vertical pink lines indicate the caustic angles at (a) 0°, 180°, (b) 5.7°, 174.3°, (c) 11.5°, 168.5°, (d) 17.5°, 162.5°.

**Figure 2:**
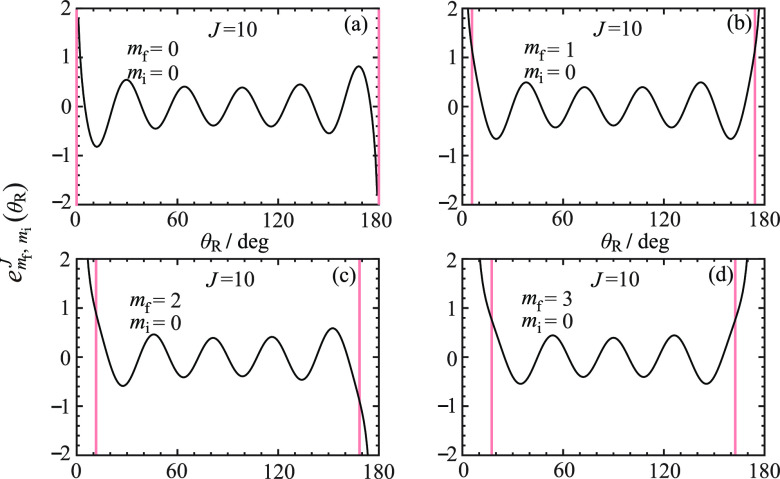
Plots of *e*_*m*_f_, *m*_i__^*J*^ (θ_R_) vs θ_R_/deg for *J* = 10, *m*_i_ = 0 (a) *m*_f_ = 0, (b) *m*_f_ = 1, (c) *m*_f_ = 2, (d) *m*_f_ = 3. The vertical pink lines indicate the caustic angles at (a) 0°, 180°, (b) 5.7°, 174.3°, (c) 11.5°, 168.5°, (d) 17.5°, 162.5°.

However, there is a problem with the unrestricted decomposition for *m*_f_ > 0. Although the ^unres^NF decomposition (5)–(7) is mathematically exact, its *physical usefulness* requires that the *d*_*m*_f_,0_^*J*^(θ_R_) and *e*_*m*_f_,0_^*J*^(θ_R_) oscillate as θ_R_ varies in the range of (0°,180°). Figures 1 and 2 examine this point by showing plots of the *little d* and *little e* functions, respectively, versus θ_R_/ deg for *J* = 10, *m*_i_ = 0, and (a) *m*_f_ = 0 (the Legendre case), (b) *m*_f_ = 1, (c) *m*_f_ = 2, (d) *m*_f_ = 3. Note that the *little d* function has *J* – *m*_f_ zeros and the *little e* function has *J* – *m*_f_ + 1 zeros. We make the following observations about Figures 1 and 2:

- We see that the *little e* function diverges as θ_R_ → 0°, 180°, which means that the N,F components of eq 6 also diverge. Then we have the unfortunate situation in the interesting forward and backward regions that σ^(N,F)^(θ_R_)→∞, whereas σ(θ_R_) → 0; i.e., although the NF decomposition (5)–(7) is mathematically exact, it is not physically meaningful at small and large angles.
- We see there are angular regions where the *little d* and *little e* functions are oscillatory (which can be called classically allowed regions) separated from two nonoscillatory regions (classically forbidden regions) when θ_R_ is close to 0°, 180°. We can distinguish between these regions using the notion of *caustics*, which are discussed next.

### 2.3 Caustic Properties of *d*_*m*_f_,0_^*J*^(θ_R_) and *e*_*m*_f_,0_^*J*^(θ_R_)

The boundaries between the two classically forbidden regions and the classically allowed region in Figures 1 and 2 can be conveniently characterized by two caustic angles,^9,10^ denoted θ_R min_^(*J*,*m*_f_)^ and θ_R max_^(*J*,*m*_f_)^, for given values of *J* and *m*_f_. They are defined by the divergence of the primitive Wentzel-Kramers-Brillouin (WKB) approximation for two linearly independent solutions of the second-order differential equation satisfied by the *little d* and *little e* functions, which become the associated Legendre differential equation because *m*_i_ = 0.

**Figure 3:**
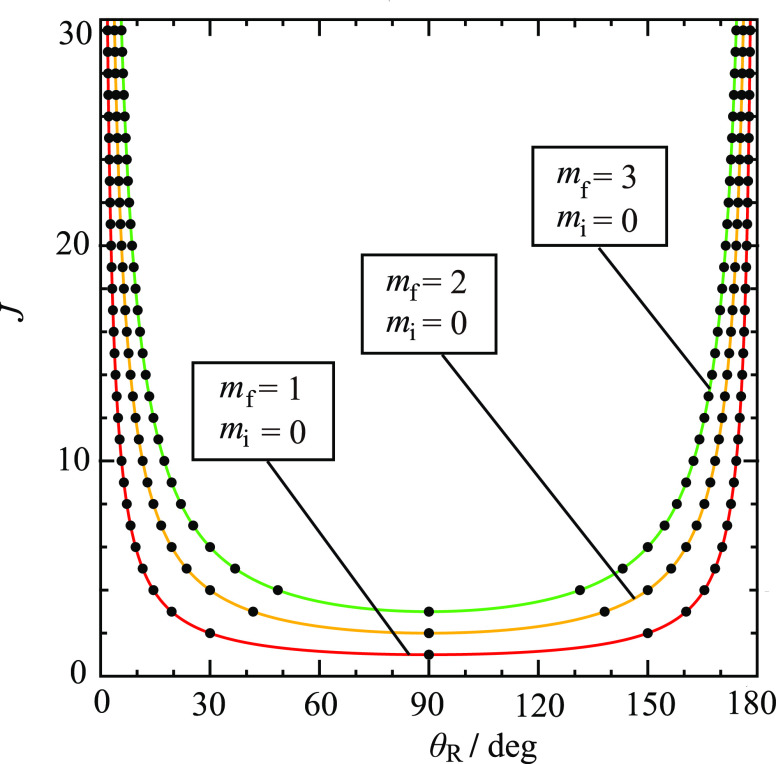
Values of θ_R min_^(*J*, *m*_f_)^/deg and θ_R max_^(*J*, *m*_f_)^/deg (black solid circles) on a (θ_R_/deg, *J*) plot for *m*_i_ = 0 and *m*_f_ = 1,2,3. Passing through the black solid circles are the curves, *J* = *m*_f_/sin θ_R_, colored red for *m*_f_ = 1, orange for *m*_f_ = 2, and green for *m*_f_ = 3.

The caustic angles can be found from eq (5.13) of ref (26), and they are determined by sin θ_R_ = *m*_f_/*J*, which results in
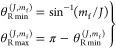
11For *J* = 10, the caustics occur at: θ_R min_^(10,*m*_f_)^ = 0° (*m*_f_ = 0), 5.7° (*m*_f_ = 1), 11.5° (*m*_f_ = 2), 17.5° (*m*_f_ = 3). The corresponding values for θ_R max_^(10,*m*_f_)^ are 180° (*m*_f_ = 0), 174.3° (*m*_f_ = 1), 168.5° (*m*_f_ = 2), 162.5° (*m*_f_ = 3). These caustic angles are marked on Figures 1 and 2 as vertical pink lines. Note that the caustics for the Legendre case (*m*_f_ = 0) are always at 0° and 180° for all values of *J* ≥ 1. The caustic angles are shown in a different way in Figure 3 on a (θ_R_/deg, *J*) plot^9,10^ for *m*_f_ = 1,2,3 and *J* = 1(1)30. This figure shows clearly that θ _R min_^(*J*,*m*_f_)^ → 0 and θ _R max_^(*J*,*m*_f_)^ → π as *J* increases for a fixed value of *m*_f_.

The above discussion implies that the *d*_*m*_f_,0_^*J*(N,F)^(θ_R_) values only exhibit an oscillatory behavior (for a given *J* and *m*_f_) in the angular range
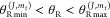
12This in turn implies that the NF decomposition (7) should work best when θ_R_ satisfies the inequality (12).

An inspection of Figure 3 shows, for given values of θ_R_, *J*, and *m*_f_, that there is a minimum value of *J*, denoted *J*_min_^(*m*_f_)^(θ_R_), such that θ_R_ satisfies the inequality (12). For *m*_f_ > 0, we have
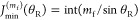
13where int(*x*) ≡ integer part of *x*. Sometimes, +1 is added to the right-hand-side of eq 13 to exclude the case where *J* = *m*_f_. In practice, it makes little difference whether +1 is added or not.^9,10^ We confirmed this is the case in our calculations for all six transitions. The physical reason is that the PWS (1) receives its main numerical contribution from partial waves with *J* ≫ *m*_f_, helped by the (2*J* + 1) factor.

The comments just given lead us to introduce the *restricted nearside-farside decomposition*, denoted ^res^NF, which we discuss next.

### 2.4 Restricted Nearside-Farside Decomposition (^res^NF)

The decomposition in which partial waves with *J* < *J*_min_^(*m*_f_)^(θ_R_) are omitted from eqs 1 and (7) defines ^res^NF.^9,10^ The restricted N,F subamplitudes are given by
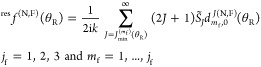
14And the corresponding ^res^NF DCSs are
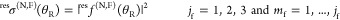
15Note that ^res^NF is an *approximate* decomposition because it omits partial waves from classically forbidden regions of θ_R_; that is, it neglects the following terms in the PWS (1)
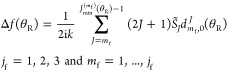
16The contribution of each partial wave in eq 16 is nonoscillatory and small in magnitude. Notice that eqs 13 and (14) are, in general, *discontinuous* functions of θ_R_. As a result, the corresponding DCSs also exhibit discontinuities, although, as we shall see in Section 5, they are usually small and confined to small and large angles. In addition, there is no global LAM for ^res^NF because of the discontinuities. Having identified the Δ*f*(θ_R_) term of eq 16, we can include it in the ^res^NF decomposition—this gives rise to the *restrictedΔ nearside-farside decomposition*, denoted ^resΔ^NF, which we discuss next.

### 2.5 RestrictedΔ Nearside-Farside Decomposition (^resΔ^NF)

The ^resΔ^NF decomposition is obtained when we combine eq 16 with eq 14 to obtain an improved version of ^res^NF. We have for the subamplitudes^7^
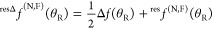
17And the corresponding ^resΔ^NF DCSs are
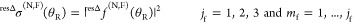
18Notice that ^resΔ^NF is an *exact* NF decomposition, unlike eq 14, which is approximate. Similar to ^res^NF, eq 17 is also a discontinuous function of θ_R_, although the discontinuities are usually small in the corresponding DCSs and confined to small and large angles—examples are provided in Section 5. In addition, there is again no global LAM for ^resΔ^NF because of these discontinuities.

*Note*: If, for a given θ_R_, we have that *J*_min_^(*m*_f_)^(θ_R_) is equal to *m*_f_, then ^unres^NF, ^res^NF, and ^resΔ^NF become equivalent.

#### Practical Remark

It can often happen that *J*_min_^(*m*_f_)^(θ_R_), for particular values of θ_R_ and *m*_f_, can exceed *J*_max_, that is, *J*_min_^(*m*_f_)^(θ_R_) > *J*_max_. For example, *J*_min_^(*m*_f_ = 3)^(θ_R_ = 0.1°) = 1718, but *J*_max_ = 40. Then many computer programs applied directly to eqs 14–(18) will crash as they attempt to use values of *S̃*_*J*_ that are undefined for *J* > *J*_max_, when the upper limit of *J* = ∞ has been replaced by *J* = *J*_max_. This problem can be avoided by adding sufficient *S̃*_*J*_ ≡ 0 to the PWS for *J* > *J*_max_.

## 3 Resummation of the Partial Wave Series

It is well-established that a resummation of a Legendre PWS can significantly improve the physical effectiveness of an NF decomposition.^13−19^ Totenhofer et al.^19^ have provided an extensive discussion of the Legendre case. This same improvement in NF physical effectiveness occurs for a basis set of *little d* functions, although this has only been studied for a single example, namely, Ar + HF rotationally inelastic scattering.^12^

It has been found previously that the biggest effect for *cleaning* the N,F DCSs and N,F LAMs of unphysical oscillations occurs on going from *resummation order*, *r* = 0 (no resummation, i.e., eq 1) to resummation order, *r* = 1. There is usually a smaller cleaning effect for further resummations, *r* = 1 to *r* = 2 and *r* = 2 to *r* = 3.

Whiteley et al.^12^ have resummed the PWS (1), which we now write as *f*_*r*__=0_(θ_R_), from *r* = 0 to *r* = 1. We do not repeat the derivation here, which exploits the recurrence relation obeyed by the *little d* functions; rather, we simply write down the final result for the resummed representation for *f*_*r*__=1_(θ_R_). From eq (3.9) of ref (12) with *m*_i_ = 0, we have
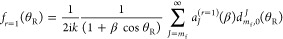
19where β ≡ β_1_ ≡ β_1_^(*r*=1)^ is the *resummation parameter*, and
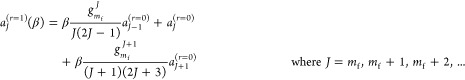
20with
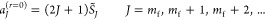
21and
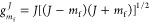
22
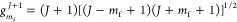
23Equation 19 also assumes that (1 + β cos θ_R_) ≠ 0. Notice that eq 20 is valid for *J* = *m*_f_, as proven in the Appendix of ref (12). For this case, we see from eq 22 that *g*_*m*_f__^*m*_f_^ = 0.

An NF decomposition of eq 19 can now be made
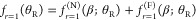
24with the corresponding N,F subamplitudes given by (θ_R_ ≠ 0,π)
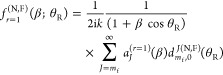
25and the corresponding N,F *r* = 1 DCSs are
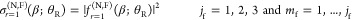
26Similar equations apply to the ^res^NF and ^resΔ^NF decompositions. For the unrestricted NF decomposition, the NF *r* = 1 LAMs are given by
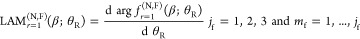
Notice that the full amplitudes, *f*_*r*__=0_(θ_R_) and *f*_*r*__=1_(θ_R_), are independent of β and numerically the same for a given value of θ_R_. This is also true for the full LAMs, LAM_*r*=0_(θ_R_) and LAM_*r*=1_(θ_R_).

In our applications, we need to choose a value for the resummation parameter β. We extend the prescription used by Anni et al.^13^ and solve the linear equation
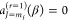
27This results in
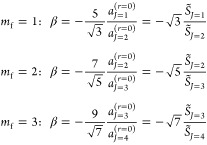
These values are used in the resummation calculations of Sections 5–7. The general result is
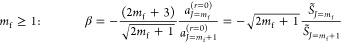


## 4 Properties of the Input Scattering Matrix Elements

We use the same S matrix elements that were computed by Yuan et al.^1^ and used for the *m*_f_ = 0 analyses in XC1.^6^ The Boothroyd-Keogh-Martin-Peterson potential energy surface number two (BKMP2) was employed.^27^ Converged S matrix elements were obtained for translational energies *E*_trans_ up to 3.5 eV. All our results are for *E*_trans_ = 1.35 eV, which is the same translational energy as that employed in the molecular-beam experiments. The masses used are *m*_H_ = 1.0078 u and *m*_D_ = 2.0141 u, with the initial translational wavenumber being *k* = 11.692 *a*_0_^–1^. For each transition, *J*_max_ is ∼40.

**Figure 4:**
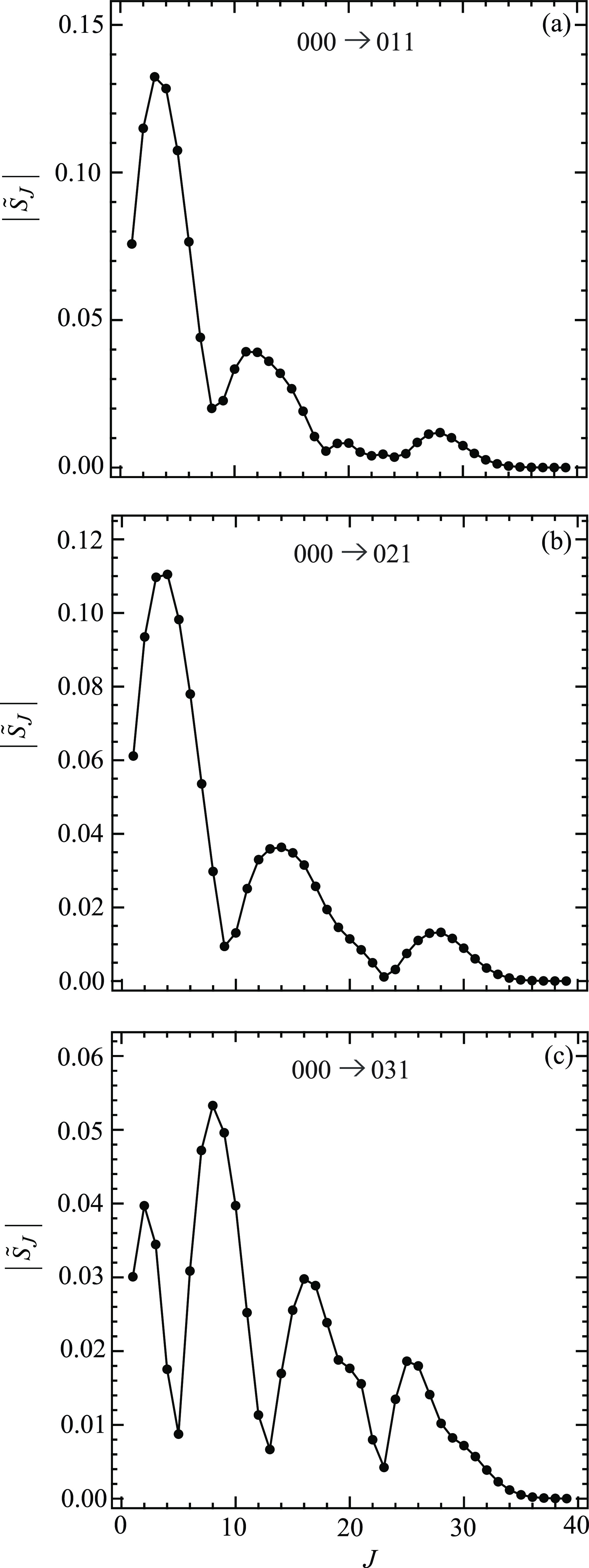
Plots of | *S̃*_*J*_ | vs *J* at *E*_trans_ = 1.35 eV. The black solid circles are the numerical S matrix data, {| *S̃*_*J*_ |}, at integer values of *J*, which have been joined by straight lines. The transitions are (a) 000 → 011, (b) 000 → 021, and (c) 000 → 031.

**Figure 5:**
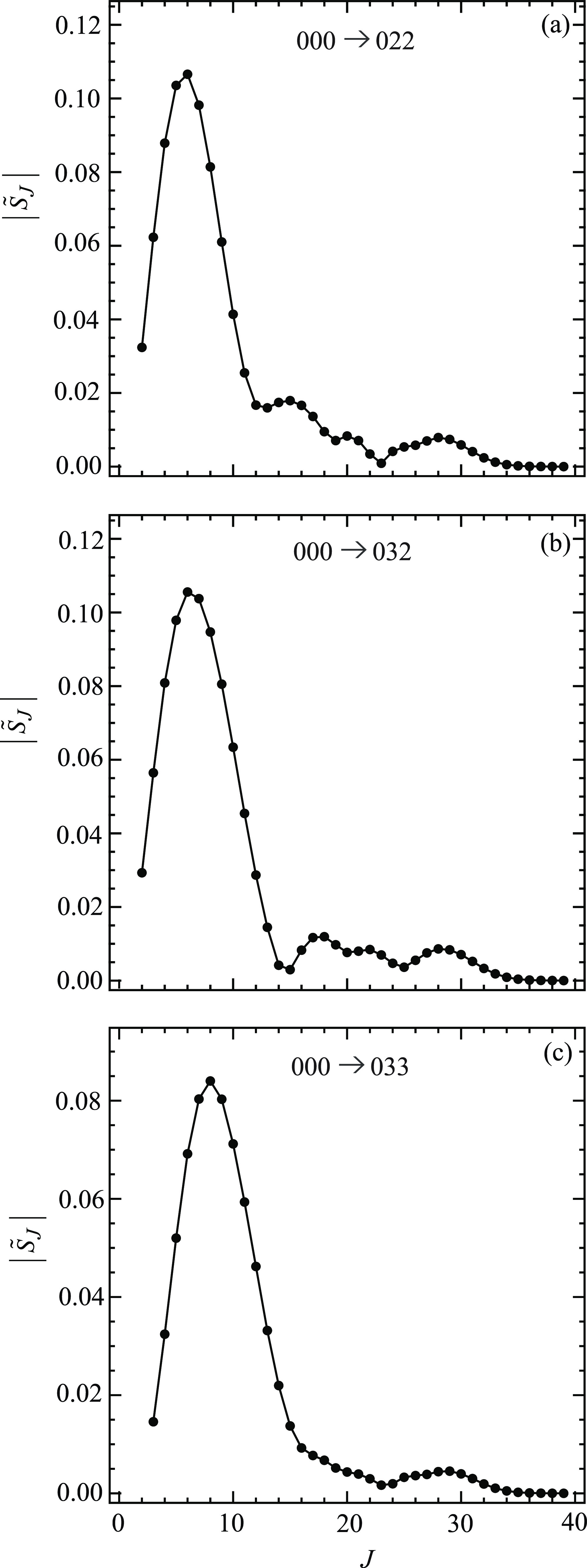
Plots of | *S̃*_*J*_ | vs *J* at *E*_trans_ = 1.35 eV. The black solid circles are the numerical S matrix data, {| *S̃*_*J*_ |}, at integer values of *J*, which have been joined by straight lines. The transitions are (a) 000 → 022, (b) 000 → 032, and (c) 000 → 033.

**Figure 6:**
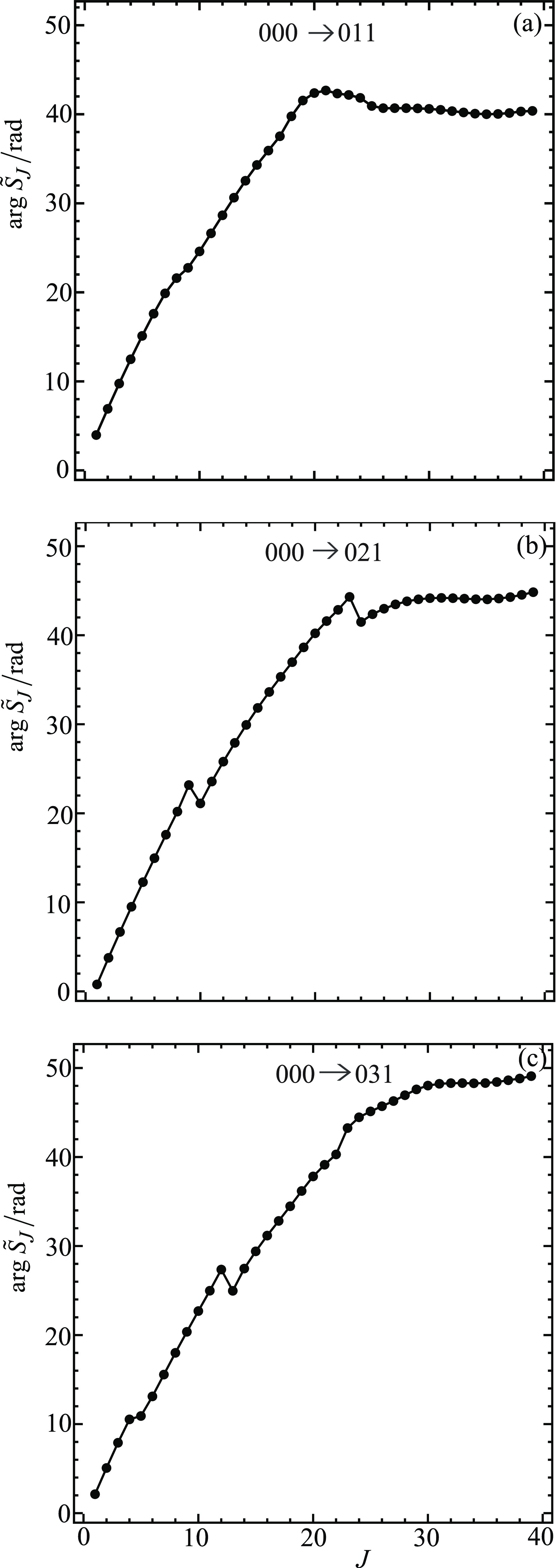
Plots of arg *S̃*_*J*_/rad vs *J* at *E*_trans_ = 1.35 eV. The black solid circles are the numerical S matrix data, {arg *S̃*_*J*_/rad}, at integer values of *J*, which have been joined by straight lines. The transitions are (a) 000 → 011, (b) 000 → 021, and (c) 000 → 031.

**Figure 7:**
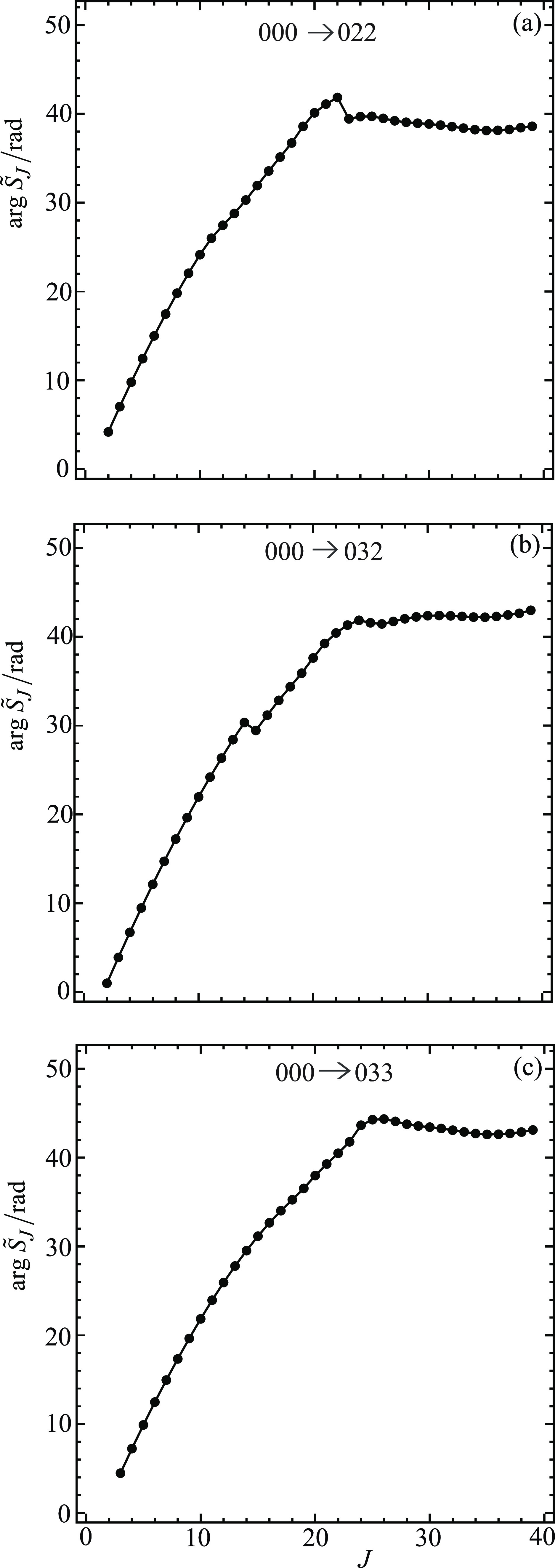
Plots of arg *S̃*_*J*_/rad vs *J* at *E*_trans_ = 1.35 eV. The black solid circles are the numerical S matrix data, {arg *S̃*_*J*_/rad}, at integer values of *J*, which have been joined by straight lines. The transitions are (a) 000 → 022, (b) 000 → 032, and (c) 000 → 033.

Figure 4 shows graphs of |*S̃*_*J*_| versus *J* for the three transitions 000 → 011, 000 → 021, 000 → 031, while Figure 5 shows plots for the remaining three transitions 000 → 022, 000 → 032, 000 → 033. Figures 6 and 7 display the corresponding plots for arg *S̃*_*J*_/rad versus *J*. Note that all the curves start at *J* = *m*_f_. A perusal of Figures 4–7 reveals the following:

- For five of the transitions, the global maximum in an |*S̃*_*J*_| plot is at the first peak as *J* increases from *J* = *m*_f_. The exception is the 031 case, where the maximum occurs at the second peak. The peaks are then followed by subsidiary local maxima; these play an important role in the interpretation of the intermediate- and large-angle scattering using the SOM in Section 8. The overall shapes of the *m*_f_ = 1,2,3 curves in Figures 4 and 5 are similar to those for the four *m*_f_ = 0 transitions, with the exception that the global maxima of the |*S̃*_*J*_| curves are always at *J* = 0 when *m*_f_ = 0 (see Figure 1 of XC1 (i.e., ref (6))).
- Figures 6 and 7 show that the plots of arg *S̃*_*J*_/rad versus *J* are roughly quadratic in shape. The *kinks* in some of the curves are seen to correspond to near-zeros in |*S̃*_*J*_|, where the phase of *S̃*_*J*_ varies more rapidly with *J*. The curves in Figures 6 and 7 have similar properties to the arg *S̃*_*J*_/rad plots for *m*_f_ = 0 (see Figure 2 of XC1 (i.e., ref (6))).
- Note that, in the NF analysis, only the values of *S̃*_*J*_ at *J* = 0,1,2,... are used. To help guide the eye, the points (black solid circles) in Figures 4–7 have been joined by straight lines. This was also done in Figures 1 and 2 of XC1.^6^ When we want a smooth continuation of the {*S̃*_*J*_} to real values of *J*, for example, for use in an asymptotic (semiclassical) analysis, we would typically use a cubic B-spline interpolation.^6^ Notice also that the kinks do not affect the NF analysis nor the asymptotic analysis, as explained in XC1.^6^

We next consider in more detail the properties of the ^unres^NF, ^res^NF, and ^resΔ^NF decompositions.

## 5 Properties of the Unrestricted, Restricted and RestrictedΔ Nearside-Farside Decompositions Including Resummations

**Figure 8:**
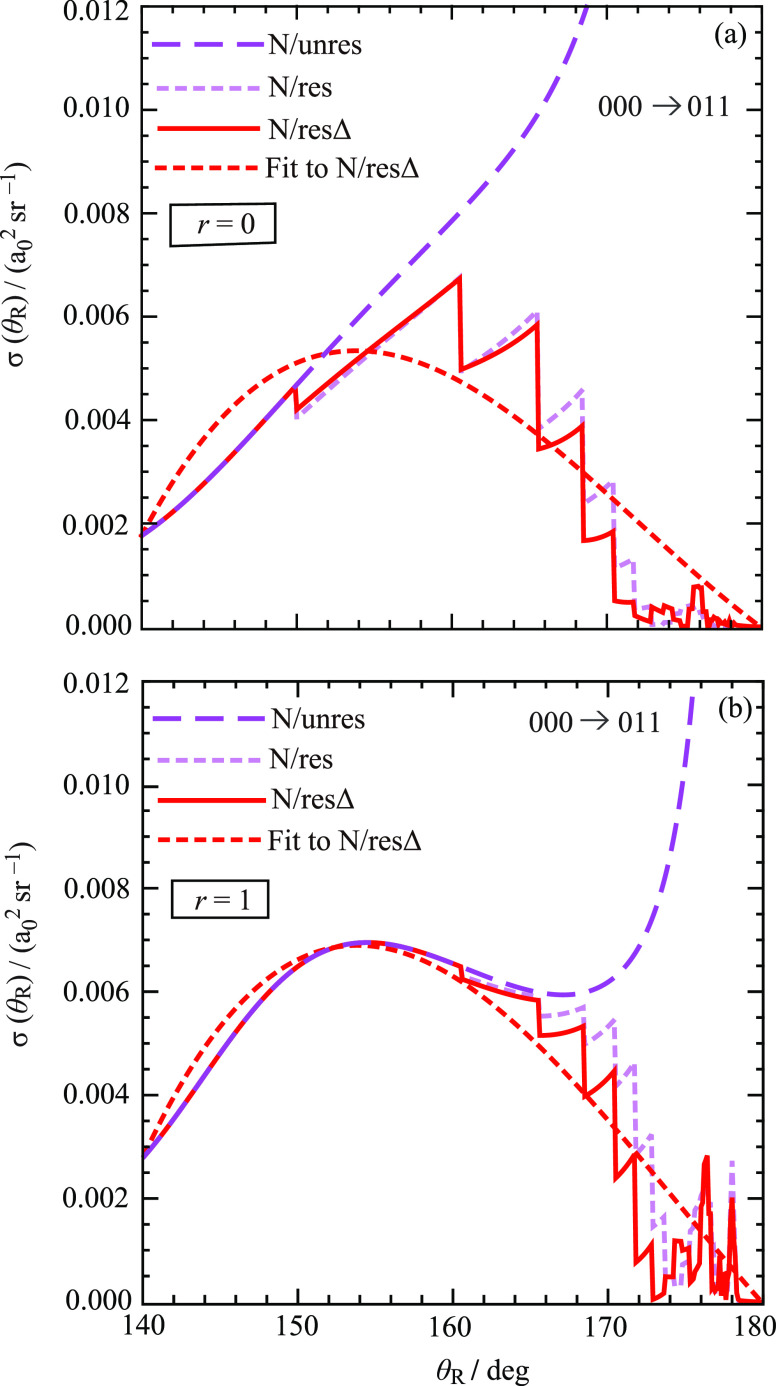
Plots of four PWS N DCSs in the large-angle region from θ_R_ = 140° to θ_R_ = 180° for the transition 000 → 011 at *E*_trans_ = 1.35 eV for (a) *r* = 0 and (b) *r* = 1. Purple long-dashed curve: PWS/N/unres. Lilac dashed curve: PWS/N/res. Red solid curve: PWS/N/resΔ. Red dashed curve: Least-squares-fit to PWS/N/resΔ.

In Sections 2.2, 2.4, and 2.5, we developed the theory for the ^unres^NF, ^res^NF, and ^resΔ^NF decompositions, respectively, for *r* = 0; the extension of the theory to *r* = 1 was given in Section 3. In the present section, we investigate in detail how these three decompositions (including resummations) influence the corresponding N,F DCSs at small and large angles, as this has not been investigated before. In Figure 8, we plot four N DCSs for the 000 → 011 transition at large angles, namely, for θ_R_ = 140^◦^–180^◦^. The upper panel shows DCSs for *r* = 0, and the lower panel shows DCSs for *r* = 1.

We begin our discussion with the ^res^N DCS (lilac dashed curve) and the ^resΔ^N DCS (red solid curve) in Figure 8. We note the following:

- By construction, the ^res^N and ^resΔ^N DCSs tend to zero as θ_R_ →180^◦^. Their discontinuities are clearly visible on the scale of the drawings. The density of jumps increases as θ_R_ →180^◦^; this is expected from Figure 3.
- The ^resΔ^N DCS is usually smaller than the ^res^N DCS for both *r* = 0 and *r* = 1. Now both the ^res^N subamplitude and the Δ*f*(θ_R_)/2 term in eq 17 are complex-valued quantities, which means that destructive interference can occur, resulting in the ^resΔ^N DCS being smaller than the ^res^N DCS.
- The first discontinuity for increasing θ_R_ occurs at θ_R_ ≈ 150° for *r* = 0 but at θ_R_ ≈ 161^◦^ for *r* = 1. This behavior can be understood because *J*_min_^(*m*_f_=1)^(θ_R_), which is equal to int(*m*_f_/sin θ_R_) by eq 13, jumps from *J* = 1 at θ_R_ ≈ 149.9° to *J* = 2 at θ_R_ ≈ 150.0°, causing a discontinuity in the PWS (14) and in the resulting ^resΔ^N and ^res^N DCSs.
  In contrast, for *r* = 1, the *J* = *m*_f_ = 1 term is put equal to zero by the choice of β in eq 27, resulting in the PWS (25) starting at *J* = *m*_f_ + 1 = 2. Then the first jump occurs for *J* = 2 at θ_R_ ≈ 160.5° to *J* = 3 at θ_R_ ≈ 160.6°.
- Because of congestion in the graphs, it is difficult for the eye to follow the jumps in the ^resΔ^N and ^res^N DCSs in Figure 8, especially as θ_R_ → 180°. However, it is the general trend in these DCSs that is of interest. We therefore made least-squares-fits to the ^resΔ^N DCSs. These are shown as red dashed curves for *r* = 0 and *r* = 1 in Figure 8a,b, respectively.

We also plotted the ^unres^N DCSs (purple long-dashed curves) for *r* = 0 and *r* = 1 in Figure 8. As expected, they tend to infinity as θ_R_ → 180°. The beneficial effect of cleaning can be seen because the ^unres^N *r* = 0 DCS starts to diverge at θ_R_ ≈ 150°, whereas for *r* = 1, the ^unres^N DCS diverges at a larger angle, namely, θ_R_ ≈ 170°.

The results discussed above for the 000 → 011 transition have all been for N DCSs at *large* angles. We also did a similar analysis for the N DCSs at *small* angles and obtained analogous results (not shown). In addition, we also calculated ^unres^F, ^res^F, and ^resΔ^F DCSs at large and small angles and obtained comparable results (also not shown). Finally, we performed ^unres^NF, ^res^NF, and ^resΔ^NF analyses for the remaining five transitions at large and small angles, again finding similar results (not shown) to the 011 case.

The least-squares-fits to the ^resΔ^NF DCSs are used in the next section, where we report NF analyses of the full DCSs for all the transitions.

## 6 Full and Nearside-Farside DCSs Including Resummations

**Figure 9:**
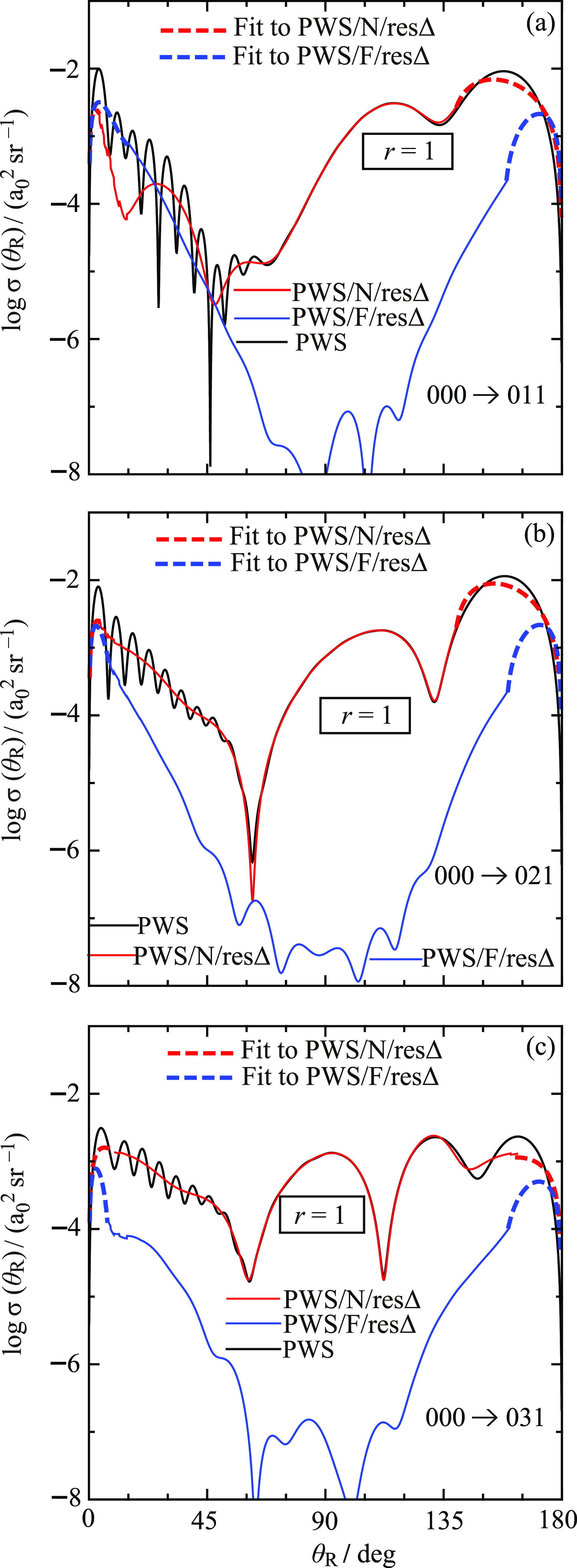
Plots of log σ(θ_R_) vs θ_R_/deg at *E*_trans_ = 1.35 eV for *r* = 1. Black curve: PWS. Red solid curve: PWS/N/resΔ. Blue solid curve: PWS/F/resΔ. Red dashed curves: least-squares-fits to PWS/N/resΔ in the small- and large-angle regions. Blue dashed curves: Least-squares-fits to PWS/F/resΔ in the small- and large-angle regions. The transitions are (a) 000 → 011, (b) 000 → 021, and (c) 000 → 031.

**Figure 10:**
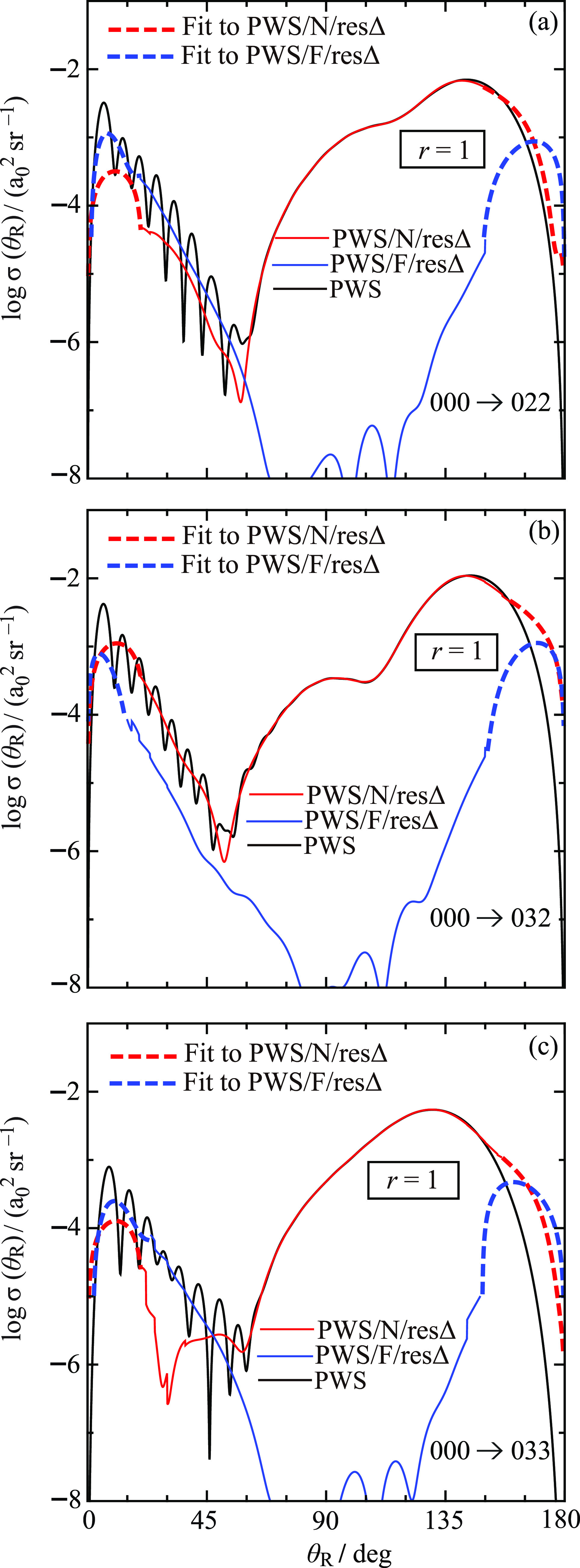
Plots of log σ(θ_R_) vs θ_R_/deg at *E*_trans_ = 1.35 eV for *r* = 1. Black curve: PWS. Red solid curve: PWS/N/resΔ. Blue solid curve: PWS/F/resΔ. Red dashed curves: least-squares-fits to PWS/N/resΔ in the small- and large-angle regions. Blue dashed curves: least-squares-fits to PWS/F/resΔ in the small- and large-angle regions. The transitions are (a) 000 → 022, (b) 000 → 032, and (c) 000 → 033.

Figure 9 shows logarithmic plots of the full and ^resΔ^N, ^resΔ^F *r* = 1 DCSs versus θ_R_ for the 000 → 011, 000 → 021, and 000 → 031 transitions. The corresponding DCSs for the 000 → 022, 000 → 032, and 000 → 033 transitions are displayed in Figure 10. For clarity of viewing, notice that, at large and small angles, least-squares-fits to the ^resΔ^N and ^resΔ^F DCSs are plotted, as explained in Section 5. We use the following color conventions for the DCSs in Figures 9 and 10 as well as in some other figures.

- Full PWS: black solid, with the label, “PWS”.
- ^resΔ^N *r* = 1 PWS: red solid, with the label, “PWS/N/resΔ”.
- Fit to ^resΔ^N *r* = 1 PWS: red dashed, with the label, “Fit to PWS/N/resΔ”.
- ^resΔ^F *r* = 1 PWS: blue solid, with the label, “PWS/F/resΔ”.
- Fit to ^resΔ^F *r* = 1 PWS: blue dashed, with the label, “Fit to PWS/F/resΔ”.

We first examine the full DCS for the 000 → 011 transition in Figure 9a. As θ_R_ increases from 0° to 180°, we observe the following.

- The DCS = 0 *a*_0_^2^ sr^–1^ at θ_R_ = 0° followed by the next observation listed here.
- Fast oscillations in an angular range extending up to θ_R_ ≈ 50°, accompanied by a decreasing DCS. This behavior merges into the next observation listed here.
- An increasing DCS with slow oscillations, which extend into the large-angle region.
- The DCS = 0 *a*_0_^2^ sr^–1^ at θ_R_ = 180°.

The full DCSs for the remaining five transitions exhibit similar properties to the 011 case and are not discussed separately. We can also compare with the four full DCSs for the *m*_f_ = 0 case shown in Figure 3 of XC1.^6^ We see that the *m*_f_ = 0 and *m*_f_ > 0 DCSs are alike, the main difference being (a) the *m*_f_ = 0 DCSs are nonzero at θ_R_ = 0°,180° unlike the *m*_f_ > 0 DCSs, (b) the angular regions separating the fast and slow oscillations are slowly varying for *m*_f_ = 0, whereas there are pronounced minima when *m*_f_ > 0.

Next, we examine the ^resΔ^N, ^resΔ^F *r* = 1 DCSs in Figures 9 and 10, making use of the exact *Fundamental Identity for Full and N,F DCSs* given by eq 9, which is also valid for the *r* = 1 case.^25^ In angular regions where there are fast oscillations, we see that the ^resΔ^N and ^resΔ^F *r* = 1 DCSs are varying relatively slowly with θ_R_, which tells us that the fast oscillations in the full DCSs arise from NF interference. Another name for the fast oscillations is Fraunhofer diffraction/oscillations. In contrast, the slow oscillations are seen to be ^resΔ^N-dominated. Thus, we have the *important result* from the NF analysis that the fast and slow oscillations arise from different physical mechanisms. This is also the case for the *m*_f_ = 0 DCSs.^6^

We can extract useful information from the periods Δθ_R_ of the fast oscillations. A simple NF model shows that these oscillations are analogous to the interference pattern from the well-known “Young’s double-slit experiment”, as explained in a molecular scattering context in Appendix A of ref (28). This analogy was also used in XC1,^6^ and it yields the simple relation
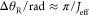
28where *J*_eff_ is an effective total angular momentum variable characteristic of the NF oscillations. For example, for the *m*_f_ = 0 DCSs, we have *J*_eff_ = *J*_g_ + 1/2, where *J*_g_ is the glory angular momentum variable, defined as the position of a local maximum in a plot of arg *S̃*(*J*)/rad versus *J* (see Figure 2 of XC1^6^). Figures 9 and 10 show that Δθ_R_ usually lies in the range of Δθ_R_ = 6°–7°, which is similar to the *m*_f_ = 0 DCSs. Then eq 28 gives *J*_eff_ = 30.0–25.7. An inspection of Figures 6 and 7 shows that these values for *J*_eff_ are also close to a local maximum in the arg *S̃*(*J*)/rad plots.

## 7 Full and Nearside-Farside LAMs Including Resummations

A full and N,F LAM analysis provides information on the value of the total angular momentum variable that contributes to the scattering at an angle θ_R_, under semiclassical conditions. An important tool^16,25^ for interpreting a LAM plot is the exact *Fundamental Identity for Full and N,F LAMs*, which is also valid for *r* = 1 and is analogous to the identity for DCSs given by eq 9.

**Figure 11:**
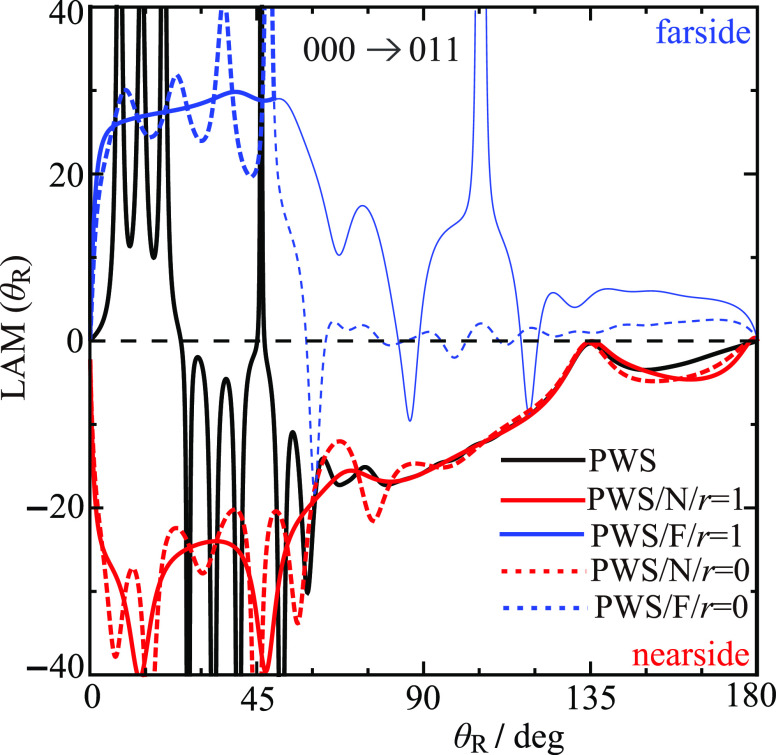
Plots of LAM (θ_R_) vs θ_R_/deg at *E*_trans_ = 1.35 eV for the 000 → 011 transition, showing results for both *r* = 0 and *r* = 1. Black curve: PWS. Red solid curve: PWS/N*/r* = 1. Red dashed curve: PWS/N*/r* = 0. Blue solid curve: PWS/F/*r* = 1. Blue dashed curve: PWS/F/*r* = 0. The fainter blue solid and dashed curves show where the F LAM(θ_R_) is not physically significant.

Figure 11 shows a full and N,F LAM plot for the 000 → 011 transition using the ^unres^NF decomposition for *r* = 0 and *r* = 1. We first make the following observations on the *full LAM*.

- The full LAM shows oscillations at small angles. At intermediate and larger angles it becomes monotonic and increases except for θ_R_ around 140°.
- The full LAM changes from F dominance to N dominance as θ_R_ increases in the small-angle region. This is the same behavior shown by the F and N *r* = 1 DCSs in Figure 9a.
- The spike at θ_R_ ≈ 46.2° corresponds to the minimum in the full DCS—see Figure 9a. Thus, the full LAM plot provides a clear indication of a change in the mechanism for the reaction as θ_R_ increases.

Next, we examine the N and F *r* = 0,1 LAMs in Figure 11 and note the following.

- The effect of cleaning the N,F *r* = 0 LAMs is very striking, with nonphysical oscillations for θ_R_ ≲ 50° being replaced by slower variations in the N,F *r* = 1 LAMs.
- It is known that N and F LAMs that are nearly zero, or oscillate about zero, are nonphysical.^13,15,16^ It can be seen in Figure 11 that this occurs for both the *r* = 0 and *r* = 1 F LAMs when θ_R_ ≳ 50°. The corresponding curves are drawn in a fainter blue compared to the F LAMs for θ_R_ ≲ 50°.
- An averaging of the N and F *r* = 1 LAMs for 10° ≤ θ_R_ ≤ 45° gives −28.1 and 28.2, respectively. The value *J* ≈ 28 is consistent with the information obtained from the periodicity of the fast oscillations in Section 6. An inspection of Figure 6a shows that *J* ≈ 28 is close to a local maximum in the arg *S̃*(*J*)/rad plot.
- The N *r* = 1 LAM for θ_R_ ≳ 50° is close to the full N LAM. And both of them monotonically increase (except for θ_R_ ≈ 140°) and are similar to the LAM for a hard-sphere collision.^13^ This implies that the SOM model, which is an approximate N theory incorporating hard-sphere dynamics, should be approximately valid at intermediate and large scattering angles. This point is confirmed in Section 8.

The properties of the full and N,F *r* = 0,1 LAMs for the other transitions are similar to those for the 011 case and are not shown separately. Overall, we can say that the information given by the LAM analysis is consistent and complementary to that in the DCS plots of Figures 9 and 10.

## 8 Semiclassical Optical Model (SOM) DCSs at Intermediate and Large Angles

The SOM is a simple procedure, introduced by Herschbach,^20,21^ for calculating the DCSs of state-to-state reactions. In XC1,^6^ we applied the SOM to the four *m*_f_ = 0 transitions and showed that the SOM provided valuable insights into structures in the DCSs at intermediate and large angles. In particular, we found that the SOM and PWS DCSs are *distorted mirror images* of the corresponding *P*_*J*_ ≡ |*S̃*_*J*_|^2^ versus *J* plots, with *J* = 0,1,2,.... The theory for the SOM has been given in XC1,^6^ and below we just state the working equations when *m*_f_ > 0.

The SOM DCS is given by
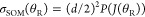
29with *P*_*J*_ ≡ *P*(*J*) and
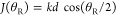
30where *J* = *m*_f_, *m*_f_ + 1,.... In eqs 29 and (30), *d* is the sum of the radii of two hard spheres representing the reactants and is the only adjustable parameter in the theory. The above equations assume that *J* ≤ *kd*; otherwise, σ_SOM_ (θ_R_) ≡ 0. Notice that the SOM only depends on the value of the modulus |*S̃*_*J*_| and is independent of arg *S̃*_*J*_. In NF terminology, the SOM is an approximate N theory, which should work best for direct rebound reactions, in particular, at intermediate and backward angles in the DCS.

**Figure 12:**
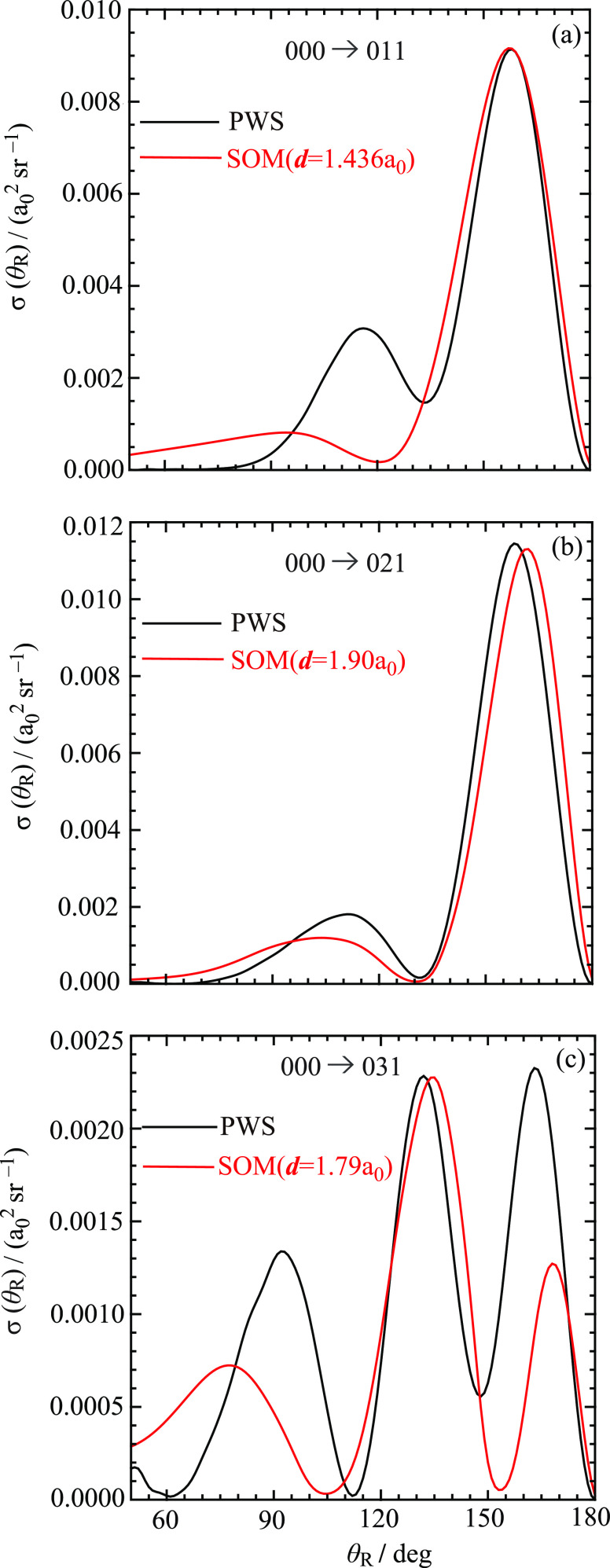
Plots of σ (θ_R_) vs θ_R_/deg at *E*_trans_ = 1.35 eV for the angular range from θ_R_ = 50° to θ_R_ = 180°. Black curve: PWS. Red curve: SOM. The transitions are (a) 000 → 011, (b) 000 → 021, and (c) 000 → 031.

**Figure 13:**
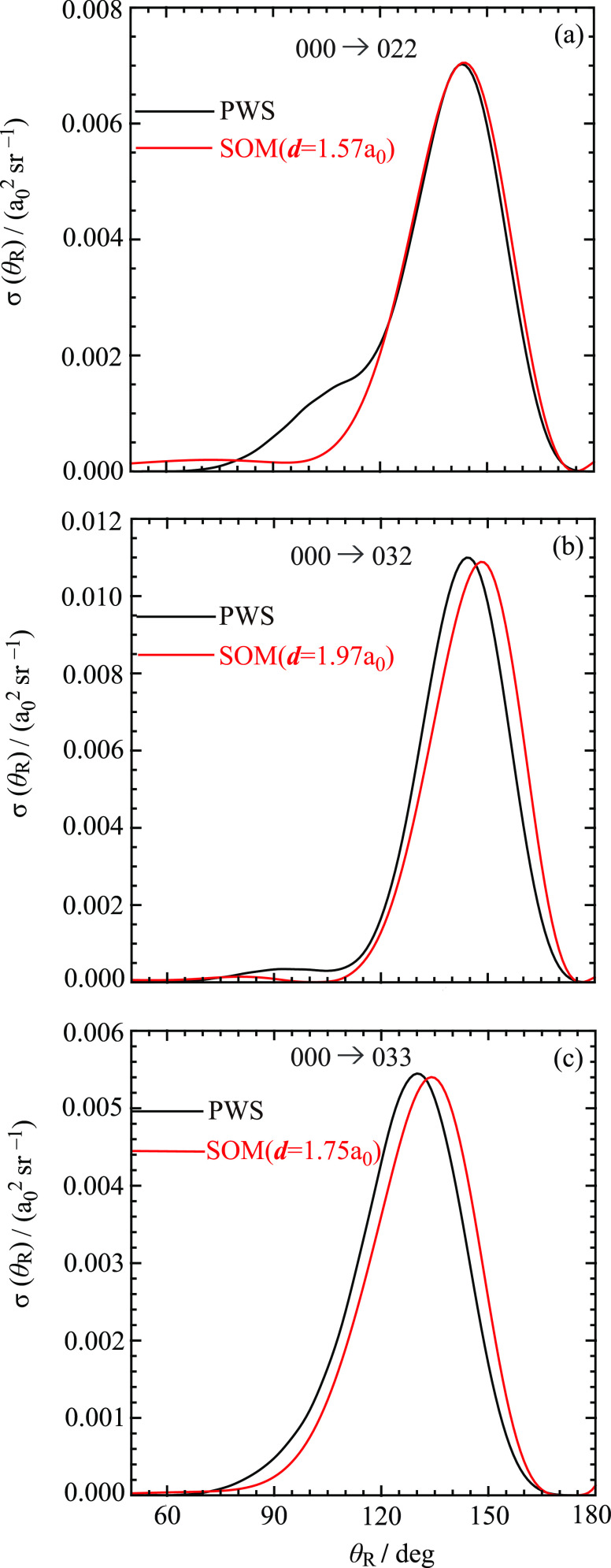
Plots of σ (θ_R_) vs θ_R_/deg at *E*_trans_ = 1.35 eV for the angular range from θ_R_ = 50° to θ_R_ = 180°. Black curve: PWS. Red curve: SOM. The transitions are (a) 000 → 022, (b) 000 → 032, and (c) 000 → 033.

The SOM and PWS DCSs are compared in Figures 12 and 13 for the six transitions in the range of θ_R_ = 50°–180°. Now, for the *m*_f_ = 0 case, we obtained values of *d* by fitting the SOM DCS to the PWS DCS at, or close to, θ_R_ = 180°. This does not work for *m*_f_ > 0 because the PWS DCSs are equal to 0 *a*_0_^2^ sr^–1^ at θ_R_ = 180°. Instead, we obtained *d* by fitting the SOM DCS at, or close to, the PWS peak nearest to θ_R_ = 180°. An exception is the 000 → 031 transition in Figure 12c, for which the second nearest peak was used (*note*, this DCS exhibits the most detailed structure out of the six transitions). The values we used for *d* are given in the figures.

It can be seen in Figures 12 and 13 that the SOM reproduces the main features in the PWS DCSs, with larger deviations as the PWS DCSs become more structured. This is encouraging, considering the simplicity of the SOM, and, like the *m*_f_ = 0 case, it tells us that the SOM and PWS DCSs are *distorted mirror images* of the corresponding *P*_*J*_ versus *J* plots. As expected, the SOM does not reproduce the NF interference (or Fraunhofer) oscillations in the PWS DCSs for θ_R_ ≲ 50° (not shown). Finally, we note that the values for *d* lie in the range of 1.44–1.97*a*_0_, which are much less than the sum of the radii at the saddle point for the BKMP2 potential energy surface, which is *d*^‡^ = *r*_HH_^‡^ + *r*_HD_^‡^ = 3.514 *a*_0_. This tells us, as was also found for *m*_f_ = 0, that the scattering at intermediate and large angles arises mainly from small values of *J* or, equivalently, from small-impact parameters.

## 9 Degeneracy Averaged Differential Cross Sections (daDCSs)

In this section, we calculate degeneracy averaged DCSs (daDCSs) and compare with the experimental daDCSs for the two transitions *v*_i_ = 0, *j*_i_ = 0 → *v*_f_ = 0, *j*_f_ = 1 and *v*_i_ = 0, *j*_i_ = 0 → *v*_f_ = 0, *j*_f_ = 3. The usual definition of a daDCS is
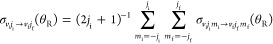
31In our applications, we have a single initial state, namely, *v*_i_ = 0, *j*_i_ = 0, *m*_i_ = 0, so eq 31 simplifies to
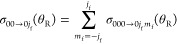
32A further simplification is possible when *m*_i_ = 0 because, as shown in Appendix A, DCSs for *m*_f_ = −1,–2,–3 are equal to those for *m*_f_ = +1,+2,+3, respectively. We can write eq 32 in the form
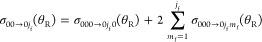
33where the sum is zero if *j*_f_ = 0. Equation 33 can also be written in a more compact way, namely
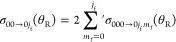
34where the prime on the Σ sign means “multiply the first term in the sum by 1/2”.

We can also substitute eqs 1 and (2) into eq 34, obtaining
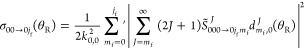
35Equation 35 is a more explicit version of eq 1 of Yuan et al.^1^

**Figure 14:**
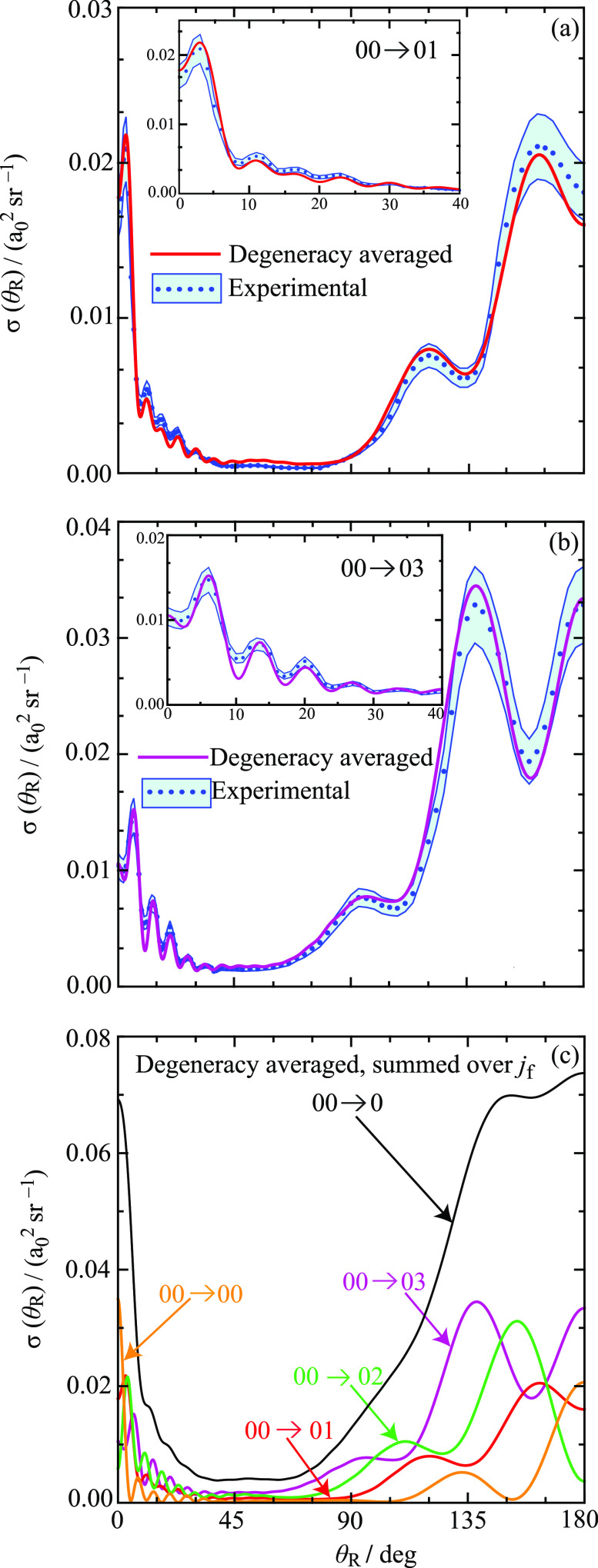
Plots of degeneracy averaged σ(θ_R_) (daDCS) vs θ_R_/deg at *E*_trans_ = 1.35 eV. (a) The transition 00 → 01 (red), together with experimental results and their estimated errors (blue). (b) The transition 00 → 03 (purple), together with experimental results and their estimated errors (blue). (c) Black curve: Degeneracy averaged, σ(θ_R_), for the 00 → 0 transition, which is summed over *j*_f_ = 0,1,2,3. The four colored curves show the degeneracy averaged σ(θ_R_) for the transitions 00 → 00 (orange), 00 → 01 (red), 00 → 02 (green), and 00 → 03 (purple).

Figure 14a compares the calculated daDCS using eq 35 for the transition 00 → 01 with the experimental data. The corresponding results for the 00 → 03 transition are in Figure 14b. A single scaling factor has been applied to the experimental data to compare with the calculations.^1^ The results in Figure 14a,b are an extension of the corresponding figures of Yuan et al.^1^ because we included *estimated* experimental uncertainties. These are a 10% error in the measurements and an angular uncertainty of 1.5°.^1^ It can be seen that the agreement between the calculated and experimental daDCSs is very good, in particular, for the NF interference (Fraunhofer) oscillations at θ_R_ ≲ 40°; these are shown in more detail in the insets. In the experiments, also note that ∼97% of the HD molecules in the molecular beam are in their ground state, and the translational energy uncertainty is ∼1.2%.^1^

If an experiment cannot resolve individual *j*_f_ states, then it is necessary to sum over these states. We have
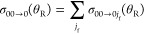
36Figure 14c shows a plot of σ_00→0_(θ_R_) versus θ_R_ over the whole angular range. It can be seen that the structure in σ_00→0_(θ_R_) (black curve) is largely washed out, even though the individual σ_00→0*j*_f__(θ_R_) in eq 36 (colored curves) possess distinct fast and slow oscillations, although less pronounced than the helicity-resolved DCSs in Figures 9 and 10. We can also relate our results and notations to the figures in the Supporting Information (SI) and main text of ref (1), with the following clarifications noted.

(a) Figure 6(c) (SI) is a dimensionless plot, as is Figure 5c (main text).^1^
(b) The three curves for *K*^′^ = 1,2,3 in Figure 5b (main text) show 2 × σ_000→03*K*′_(θ_R_).^1^
(c) The units for the labels on the ordinates of Figures 6(a), 6(b), and 7 (SI) are *a*_0_^2^ sr^–1^.^1^
(d) The red curve for *K*^′^ = 1 in Figure 6(b) (SI) shows 2 × σ_000→011_(θ_R_).^1^
(e) The red curve for *K*^′^ = 1 in Figure 7(c) (SI) shows 2 × 10^3^× [σ_000→011_(*J*_max_,θ_R_) – σ_000→011_(*J*_max_ −1,θ_R_)] versus *J*_max_ at θ_R_ = 4°.^1^ Here *J*_max_ makes explicit the finite upper value for the PWS when used in eqs 1 and (2).
(f) The blue curve for *K*^′^ = 0 in Figure 7(c) (SI) shows 10^3^× [σ_000→010_(*J*_max_,θ_R_) – σ_000→010_(*J*_max_ −1,θ_R_)] versus *J*_max_ at θ_R_ = 0.4°, not 4°.^1^
(g) The three curves for *K*^′^ = 1,2,3 in Figure 7(d) (SI) show 2 × 10^3^× [σ_000→03*K*′_(*J*_max_,θ_R_) – σ_000→03*K*′_(*J*_max_ – 1,θ_R_)] versus *J*_max_ at θ_R_ = 6°.^1^
(h) The blue curve for *K*^′^ = 0 in Figure 7(d) (SI) shows 10^3^ × [σ_000→030_(*J*_max_,θ_R_) – σ_000→030_(*J*_max_ – 1,θ_R_)] versus *J*_max_ at θ_R_ = 6°.^1^

## 10 Conclusions

We have theoretically analyzed structures in the DCSs of the ground-state reaction H + HD → H_2_ + D for the product states 011, 021, 031, 022, 032, and 033. The calculations extend and complement our previous analyses in XC1^6^ for the cases 000, 010, 020, and 030, making 10 DCSs in all. The motivation comes from the experiments and simulations of Yuan et al.,^1^ who have measured for the first time fast oscillations in the small-angle region of the daDCSs for *j*_f_ = 1 and 3 as well as slow oscillations in the large-angle region.

Our main theoretical tools were two variants of Nearside-Farside theory: (1) We applied unrestricted, restricted, and restrictedΔ NF decompositions, including resummations, to the helicity PWS, which is expanded in a basis set of *little d* functions. We analyzed in detail the properties of restricted and restrictedΔ NF DCSs and showed that they correctly go to zero in the forward and backward directions when *m*_f_ > 0, unlike the unrestricted NF DCSs, which incorrectly go to infinity. We also calculated LAMs to obtain further insights into the reaction dynamics. Properties of *little e* functions played an important role in the NF analysis, as do the caustics associated with the *little d* and *little e* functions. (2) We applied an approximate N theory at intermediate and large angles, namely, the Semiclassical Optical Model.

We showed that the fast oscillations at small angles (sometimes called Fraunhofer diffraction or oscillations) arise from an NF interference effect. In contrast, the slow oscillations at large angles are an N effect and arise in the DCS as a distorted mirror image of the corresponding *P*_*J*_ versus *J* plot. We also compared with the experimental daDCSs, obtaining very good agreement.

Our analyses confirm the earlier insight of Dobbyn et al.^7^ that as the PWS increases in complexity, this has little impact on the physical insight provided by an NF analysis.

## Appendix A

This Appendix proves the following identity for PWS DCSs when *m*_f_ = 1,2,3,...,*j*_f_ (the identity is trivially true for *m*_f_ = 0).

A1

That is, the DCSs for *m*_f_ and −*m*_f_ are equal when *m*_i_ = 0. Now the vibrational quantum numbers *v*_i_ and *v*_f_ do not change in the following, so they will be omitted from now on to simplify the notation. Note that all quantum numbers are integers.

We begin by writing the *helicity* or *body-fixed* S matrix element *S̃*_*j*_i_*m*_i_ → *j*_f_*m*_f__^*J*^ as a linear combination of *space-fixed* S matrix elements, which are labeled by *J*, *j*_i_, *j*_f_ and by *l*_i_, *l*_f_, the initial and final orbital angular momentum quantum numbers, respectively. We have^9,22,23^

A2

where ⟨*j*_1_*m*_1_, *j*_2_*m*_2_| *jm*⟩ is a Clebsch-Gordan coefficient with *m* = *m*_1_ + *m*_2_.

For *m*_i_ = 0, eq (A2) simplifies to

A3

We next replace *m*_f_ > 0 by −*m*_f_ < 0 in eq (A3) to obtain

A4

Now we have the relation [ref (24), page 42, eq (3.5.17)]

so eq A4 becomes

A5

Next we note, from ref (24), page 46, eq (3.7.3), and page 49, eq (3.7.14), that ⟨*j*_i_ 0, *J* 0 | *l*_i_0 ⟩ is zero unless *j*_i_ + *J* + *l*_i_ = an even number, which means we can introduce a factor of +1 = (−1)^*j*_i_+*J*+*l*_i_^ into eq (A5). In addition, there is the conservation of parity relation, (−1)^*j*_i_+*l*_i_^ = (−1)^*j*_f_+*l*_f_^, so for the phase factor we find (−1)^*j*_f_+*J*+*l*_f_^ = (−1)^*j*_i_+*J*+*l*_i_^ = +1 and obtain

A6

Comparing the right-hand sides of eq A3 and (A6) we see they are the same, so

A7

Now the PWS (1) for the scattering amplitude remains valid for negative helicities, provided the starting value of the summation is replaced by *J* = |*m*_f_|. When *m*_f_ > 0 is replaced with −*m*_f_ < 0, in eq 1 we get

Finally with the help of the relation [ref (24), page 60, eq (4.2.5)]

together with the result, eq (A7), we find that the moduli of the scattering amplitudes for *m*_f_ and −*m*_f_ are equal, which then gives us the identity, eq (A1).

## Appendix B

In the development and application of NF theory (NFology), it essential to use unambiguous and consistent definitions for the special functions (of the first and second kinds) used in the various NF decompositions. Here we give the precise mathematical definitions of the functions that we use, since there is often more than one convention in the literature.

For the *little d* function, which is also known as a *reduced rotation matrix element* (of the first kind) or *Wigner function*, we use the definition from ref (24), page 58, eq (4.1.23).

B1

The definition of *P*_*J*–*m*_f__^(*m*_f_,*m*_f_)^(cos θ_R_), a *Jacobi polynomial*, has become standardized (ref (24), page 57). Note that the Jacobi polynomial in eq (B1) is a special case of a *Jacobi function of the first kind.*(29) Important special values are

where δ_*m*_f_ 0_ is a Kronecker delta function.

For the *little e* function, which is also called a *reduced rotation matrix element of the second kind*, we use^10^

B2

where **Q**_*J*–*m*_f__^(*m*_f_, *m*_f_)^(cos θ_R_) is a *Jacobi function of the second kind*, which is a second independent solution of the Jacobi differential equation. We use Szegö’s definition as given in ref (29), page 78, eq (4.62.9). Note that **Q**_*J*–*m*_f__^(*m*_f_, *m*_f_)^(cos θ_R_) is defined “on the cut”, −1 < cos θ_R_ < + 1.

Since *m*_i_ = 0 in our applications, we also have the following important simplifications. From ref (24), page 59, eq (4.1.24), we get

B3

and from section 2 in the Appendix of ref (10), we have

B4

In eqs B3 and (B4), *P*_*J*_^*m*_f_^(cos θ_R_) and *Q*_*J*_^*m*_f_^(cos θ_R_) are *Ferrers’ associated Legendre functions of the first and second kinds*, respectively.^30^ They are defined by (ref (24), page 22, eq (2.5.10)).

B5

The *P*_*J*_(cos θ_R_) in eq (B5) is a *Legendre polynomial of degree J*, which is a special case of a Legendre function of the first kind of degree *J*. The *Q*_*J*_(cos θ_R_) in eq (B5) is a *Legendre function of the second kind of degree J*. Note: the other definition in common use, but not used in this paper, is that of Hobson, in which the right-hand-side of eq (B5) is multiplied by (−1)^*m*_f_^.

We also have the following simple results

and

The following asymptotic approximations are frequently useful.^7,10^ They are valid for a fixed value of *m*_f_, *J* → ∞, and 0 < θ_R_< π.

and
